# The Infinity Loop of Healthcare Innovation: Development of an Integrated Rehabilitation Pathway for Lumbar Fusion Surgery Through Design Thinking

**DOI:** 10.5334/ijic.7765

**Published:** 2025-05-12

**Authors:** Liedewij Bogaert, Bart Depreitere, Sanne Peters, Tinne Thys, Simon Brumagne, Sebastiaan Schelfaut, Koen Peers, Lieven Moke, Wim Dankaerts, Peter Van Wambeke, Ann Spriet, Thijs Willem Swinnen, Lotte Janssens

**Affiliations:** 1 Department of Physical and Rehabilitation Medicine, University Hospitals Leuven, Leuven, Belgium; 2 REVAL Rehabilitation Research, Hasselt University, Diepenbeek, Belgium; 3 Department of Neurosurgery, University Hospitals Leuven, Leuven, Belgium; 4 Department of Implementation Science, University of Melbourne, Australia; 5 Department of Rehabilitation Sciences, KU Leuven, Leuven, Belgium; 6 Institute for Research and Training (IORT), Department of Development and Regeneration, KU Leuven, Leuven, Belgium; 7 Department of Orthopaedics, University Hospitals Leuven, Leuven, Belgium; 8 Department of Quality Management, University Hospitals Leuven, Leuven, Belgium

**Keywords:** guideline, integrated care, co-creation, pathway, design thinking, co-design

## Abstract

**Introduction:**

Integrated care pathways may help to bridge evidence-practice gaps. To overcome the limitations of traditional researcher-centred and linear pathway development frameworks, a more user-centred approach is needed. In this study, we propose design thinking as a framework for developing integrated care pathways, specifically targeting rehabilitation of patients undergoing lumbar fusion surgery.

**Description:**

From 2017 to 2022, we utilized the design thinking infinity loop to create an evidence-based rehabilitation pathway for patients undergoing lumbar fusion surgery. This approach consisted of five phases: (1) empathizing with user needs, (2) defining problem statements, (3) ideating through meta-analysis, expert consensus, and brainstorming, (4) prototyping the pathway, and (5) testing its effectiveness and implementability.

**Discussion:**

Through the proposed design thinking phases, innovative elements such as prehabilitation, early mobilization, and consistent communication emerged as the building blocks of the new rehabilitation pathway, addressing the needs of both patients and healthcare providers. These results serve as a practical guide for applying design thinking in developing integrated care pathways.

**Conclusion:**

Design thinking, represented by the infinity loop, presents a user-centred framework for developing integrated care pathways, and has the potential to effectively bridge the gap between evidence and clinical practice.

## Introduction

Lumbar fusion surgery is a common surgical procedure to treat persistent radicular pain and disability caused by nerve root compression, or when low back pain is caused by gross instability of the vertebrae, consistent with radiological findings, and not improving with multimodal conservative treatment [1]. Historically, research in the field of lumbar fusion surgery has focused primarily on technical advancements, resulting in higher structural success rates in terms of bony fusion, decompression and sagittal alignment [2]. Unfortunately, clinical success rates following lumbar fusion surgery are often suboptimal, with up to 40% of patients experiencing persistent pain, lacking functional improvement, and expressing general dissatisfaction [3,4,5,6]. In addition, disappointing return-to-work rates add to the already high socio-economic burden [6]. In the case of well-indicated lumbar fusion surgery, this discrepancy between structural and clinical outcomes raises the critical concern: inadequate incorporation of optimal rehabilitation into current practice.

Despite the increasing recognition over the past two decades that rehabilitation effectively improves patient outcomes, rehabilitation tends to be overlooked in routine clinical practice [7,8,9,10,11,12]. Only one-third of patients receive preoperative rehabilitation before undergoing lumbar fusion surgery, and referral rates to postoperative rehabilitation vary widely, ranging from 44% to 88% [10,11,12]. Moreover, we have shown that unsupported practices such as prescribing bracing after lumbar fusion surgery are still being performed by up to 52% of surgeons in Belgium [13]. Another concern is the considerable variability in the timing or content of rehabilitation of patients undergoing lumbar fusion surgery [7,10,11,12,13]. Surgeons impose substantial heterogeneity in terms of activity restrictions, ranging from complete avoidance of activities such as jumping or running to no restrictions at all [12].

Rehabilitation for lumbar fusion surgery shares many principles with other types of lumbar surgery [8,14]. However, early rehabilitation after lumbar fusion is uniquely challenging due to prevalent misconceptions. For example, 42% of surgeons prescribing a brace do so based on the belief that immobilisation will improve the fusion rate. Patients often resist early mobilisation and are concerned that the surgical implants may ‘move or break and cause them harm’ [15,16,17].

As a result, lumbar fusion surgery care faces two key challenges in translating the best available evidence into clinical practice: 1) underuse of effective interventions, and overuse of ineffective interventions, and 2) gaps in the existing evidence and a lack of consensus adding to clinical variability.

To address these challenges, an evidence-based rehabilitation pathway that has the potential to be implemented in clinical practice is urgently needed. However, traditional approaches to pathway development often overlook patient and provider perspectives, resulting in solutions that inadequately address real-world clinical complexities [18]. To overcome these shortcomings, we propose using design thinking – a user-centered methodology that involves future users as co-designers – to develop an optimal integrated rehabilitation pathway for lumbar fusion surgery [19,20,21]. Originally rooted in engineering and architecture, design thinking demonstrated in healthcare to result in better satisfaction, usability and effectiveness compared to traditional methods [18,22].

Therefore, the aim of this study was to adopt a design thinking framework to establish an evidence-based, integrated rehabilitation pathway for patients undergoing lumbar fusion surgery.

## Methods

### Infinity loop of Design Thinking

#### Definitions

For the term ‘integrated care pathway’, we adopted the definition as proposed by Lawal et al. [23]. An integrated care pathway is described as a structured interdisciplinary care plan with the following characteristics: “(i) it is used to translate guidelines or evidence into local structures, (ii) it details the steps in the course of treatment or care in a plan, pathway, algorithm, guideline, protocol or other “inventory of actions” (i.e. the intervention has time-frames or criteria-based progression), (iii) it aims to standardize care for a specific clinical problem, procedure or episode of healthcare in a specific population.”

Following the most recent definition from Cochrane Rehabilitation, ‘rehabilitation’ is defined as “a multimodal, person-centred, collaborative process, including interventions targeting a person’s capacity and/or contextual factors related to performance with the goal of optimizing the functioning of persons with health conditions currently experiencing disability or likely to experience disability” [24].

We propose the term ‘integrated rehabilitation pathway’ to incorporate both concepts, integrated care pathway and rehabilitation, respectively.

#### End-users and Experts group

An interdisciplinary and interuniversity steering committee was assembled to oversee the rehabilitation pathway design. The committee consisted of twelve experienced clinicians and researchers who were purposeful selected based on their extensive expertise, with the goal of obtaining variability towards disciplines. All members were affiliated to an academic hospital (i.e. are potential end-users) and/or to a university. The steering committee was supported by a group of end-users and experts, including patients, who provided relevant expertise to the design of the rehabilitation pathway (Table 1).

**Table 1:** Professional background of (A) the steering committee; and (B) of the end-users and experts supporting the steering committee.


A) MEMBERS STEERING COMMITTEE: PROFESSIONAL BACKGROUND	NO.

Orthopaedic surgery (research and clinical)	2

Neurosurgery (research and clinical)	1

Physical and Rehabilitation Medicine (clinical)	1

Physical and Rehabilitation Medicine (research and clinical)	1

Musculoskeletal physiotherapy (clinical)	2

Musculoskeletal physiotherapy (research and clinical)	3

Musculoskeletal physiotherapy (research)	1

Process management (research and hospital management)	1

**B) SUPPORTING END-USERS AND EXPERTS: PROFESSIONAL BACKGROUND**	**NO.**

Musculoskeletal physiotherapy (clinical)	4

Psychology with expertise in cognitive behavioural therapy (research and clinical)	2

Psychomotor therapy (clinical)	2

Occupational therapy (clinical)	2

Patients	2

Neurosurgery (research and clinical)	1

Pathway development (research)	1

Implementation science (research)	1

IT and communication department	2

Clinical support management (clinical)	1


Informed consent was obtained from all participants prior to participation, and ethical approval was obtained from the Ethics Committee Research UZ/KU Leuven (S60109).

#### Design thinking process

**Figure 1:**
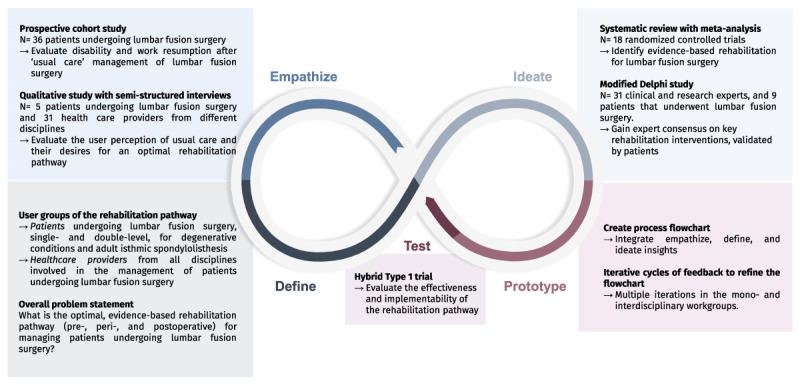
Visual summary of the design thinking infinity loop for developing an integrated rehabilitation pathway for patients undergoing lumbar fusion surgery.

From November 2017 to March 2022, we adopted the infinity loop of design thinking, a revisualization of Stanford’s d.school framework, to design an evidence-based, integrated rehabilitation pathway for patients undergoing lumbar fusion surgery [20,25]. The infinity loop includes five phases, which are non-linear (back-and-forth is possible), continuous (“trying to solve a problem can help you better understand it”), and user-centred: (i) Empathize, (ii) Define, (iii) Ideate, (iv) Prototype, and (v) Test. A visual summary of the design thinking process is provided in Figure 1.

This design thinking framework was strengthened by incorporating sound research methodologies from theoretical pathway and implementation frameworks [26,27,28,29,30,31]. The design thinking process was reported in line with the ‘Guideline for reporting research involving design’ and the GUIDED statement [32,33].

The aims, methods, and the key results of each phase of the infinity loop of design thinking are presented below. The results of the testing phase are outside the scope of this paper.

### Empathize

The empathize phase aims to understand future end-users within the context of our design challenge: understanding their actions, motivations, needs, thoughts, and what is meaningful to them. This understanding is achieved by observing and engaging with end-users directly.

To accomplish this, two studies were conducted: a prospective cohort study exploring usual care and a qualitative descriptive interview study encompassing interviews with both patients and healthcare providers.

First, we observed the usual care of 36 patients undergoing lumbar fusion surgery and their clinical outcomes until one year postoperatively in a prospective cohort study [34]. Patients were aged between 18 and 75 years old, underwent single or double-level lumbar fusion surgery for degenerative conditions, and were followed-up until one year postoperative.

The prospective cohort study observed that patients experienced an improvement of back and leg pain following lumbar fusion surgery. However, a high score for kinesiophobia and disability remained present. One year postoperatively, only 56% of patients returned to work. This highlights the (unspoken) needs for tackling these underwhelming work resumption and high disability and kinesiophobia after lumbar fusion surgery [34].

In parallel, we engaged with patients and healthcare providers, in a qualitative interview study to gain a deeper understanding of how they perceive the current practices of lumbar fusion surgery and its rehabilitation, and what ingredients are deemed necessary for an optimal rehabilitation pathway from an end-user perspective [35]. Five patients who underwent lumbar fusion surgery and 31 healthcare providers from relevant disciplines participated in these semi-structured interviews. Healthcare providers were purposeful selected to ensure diverse expertise and work environments (academic and non-academic hospitals). Patients were purposeful selected from the orthopaedic and neurosurgery department within the academic hospital to capture varied patient demographics and clinical outcomes. The interview guide was reviewed by all members of the steering committee to ensure comprehensibility and relevance of the questions and refined during the interview process. The final interview guide covered open-ended questions regarding opinions on the current and optimal rehabilitation. Interviews were audio-recorded and transcribed verbatim. Qualitative analysis of the interviews followed the QUAGOL methodology and used NVivo software [36,37].

The interviews revealed that healthcare providers disagreed on restrictions of postoperative activities, the optimal timing (preoperative, early or late postoperative) and content of postoperative physiotherapy, as well as the involvement of other disciplines in the rehabilitation process, thereby underlining the lack of consensus in current rehabilitation practice [35]. Additionally, patients and healthcare providers underlined the importance of interdisciplinary collaboration and an easy point of contact in the rehabilitation of patients. Within the interdisciplinary work setting, some healthcare providers perceived a professional hierarchy that restricted their ability to express their opinions freely [35].

Patients, on the other hand, encountered varying viewpoints from different healthcare providers, which generated uncertainty regarding their rehabilitation process and instilled fear regarding permitted movements. Importantly, both healthcare providers and patients did agree on the need for consensus amongst healthcare providers to ensure uniform messages towards patients [35].

### Define

The define phase aims to formulate a meaningful and actionable problem statement, based on the insights into the spoken and unspoken needs and understanding of end-users during the empathize phase. The steering committee discussed and defined a specific problem statement for each end-user group (i.e., patients and healthcare providers) (Table 2). Novel elements in this problem statement after the empathize phase were the timeframe covered by, and characteristics of the needed rehabilitation pathway.

**Table 2:** Problem statements.


USER GROUPS	PROBLEM STATEMENT

Patients undergoing single- and double level lumbar fusion surgery for degenerative conditions	Patients need tailored rehabilitation in the pre-, peri- and postoperative period, provided by an interdisciplinary team, to improve clinical and work-related outcomes after surgery.

Healthcare providers from all disciplines involved in the management of patients undergoing lumbar fusion surgery	Providers need an easy-to-use, evidence-based, interdisciplinary, and transmural pathway to help streamline optimal surgical rehabilitation

**Overall problem statement:** “What is the optimal, evidence-based, rehabilitation pathway (pre- peri and postoperative) for managing patients undergoing lumbar fusion surgery?”	


### Ideate

The ideate phase aims to generate innovative ideas, based on the insights of the previous phases, and will provide the fuel and source material for building prototypes in the next phase.

To lay the foundation for an evidence-based rehabilitation pathway, two distinct research methods were employed. First, a systematic review and meta-analysis were conducted to comprehensively summarize the current scientific evidence on rehabilitation interventions that could enhance the clinical outcomes of patients undergoing lumbar fusion surgery [7].

The systematic literature search identified 18 randomized controlled trials, including 1402 unique patients, comparing rehabilitation interventions in the preoperative or postoperative period of lumbar fusion with usual care. Exercise therapy was found more effective in reducing disability and pain in the short-term (standardized mean difference [95% CI]: –0.41 [–0.71; –0.10] and –0.36 [–0.65; –0.08], respectively). If this was embedded in a multimodal rehabilitation program, a greater reduction in disability and fear avoidance was observed, compared to exercise alone (–0.31 [–0.49; –0.13] and –0.64 [–1.11; –0.17], respectively). Rehabilitation showed a positive tendency towards a higher return-to-work rate, compared to usual care (pooled relative risk [95% CI]: 1.30 [0.99; 1.69]. Therefore, we could conclude that an optimal rehabilitation pathway should likely be multimodal [7].

Second, we conducted a modified Delphi study comprising three iterative rounds and one face-to-face meeting [38]. We assembled an interdisciplinary expert panel consisting of 31 experts from Belgium and the Netherlands. Experts were purposeful selected based on their extensive clinical and research experience in the domains of low back pain, rehabilitation, and lumbar fusion surgery. Efforts were made to ensure a diverse representation of disciplines, gender, and primary work setting. The first round was based on the insights during the previous phases [34,35], and on the results of the systematic review [7]. A predefined consensus threshold of 75% agreement was established a priori. In cases where consensus was not achieved, key interventions were rephrased based on experts’ feedback and reconsidered in subsequent rounds. Experts could also suggest additional key interventions to be included in subsequent rounds. The final expert consensus was validated by nine patients who underwent lumbar fusion surgery. These patients represented diverse demographic and outcome characteristics and were invited to participate in an online survey encompassing statements relevant to their experiences. Furthermore, seven of these patients engaged in a subsequent focus group, where they openly shared their experiences throughout the preoperative, perioperative, and postoperative phases. Full details of both studies are available elsewhere [7,38].

The modified Delphi study resulted in 122 key recommendations for optimal pre-, peri- and postoperative rehabilitation, endorsed by experts and validated by patients [38]. These recommendations include innovative components such as prehabilitation, early postoperative mobilization, easy access to a case manager, uniform communication through leaflets, videos and website, and limited postoperative activity restrictions. An explicit need for return-to-work guidance was formulated during the patient validation of the recommendations [38].

### Prototype

The prototype phase aims to transform ideas generated in the previous phase into tangible prototypes that allow designers to visualize and refine possible solutions.

An initial prototype was developed based on the end-users’ needs (empathize phase), rehabilitation pathway needs (define phase), and the building blocks from best-evidence, expert-consensus, and the feedback of patients (ideate phase). For this, the rehabilitation recommendations that gained expert-consensus during the modified Delphi study were categorized into key interventions and subcomponents. These key interventions were clustered into three time periods: the pre-, peri- and postoperative phase, and translated into a pathway. This was presented in the form of a time-task flowchart using Visio software, adhering to the principles of process mapping [39].

This time-task flowchart was first piloted in the interdisciplinary steering committee, where gaps and areas needing improvement were defined. Second, parts of this prototype (‘small scale prototypes’) were discussed separately on an iterative basis with the relevant supporting experts, in mono- or interdisciplinary groups. For example, the postoperative restrictions were discussed separately with the surgeons, then separately with the physiotherapists, and finally with surgeons, physiotherapists and specialists in physical and rehabilitation medicine together. These discussions started with a brainstorming session (i.e., iterative prototyping, defining areas needing improvement or additional needs and ideating new possibilities when an idea fails) and moved forward until consensus within the small supporting groups was obtained (i.e., prototyping a part of the rehabilitation pathway). Third, the final rehabilitation pathway prototype (‘full scale prototype’) was once more critically evaluated and adapted by the interdisciplinary steering committee.

As part of this process, various design supporting tools were developed for the newly developed rehabilitation pathway. These included an educational leaflet, an information leaflet for physiotherapists, an information leaflet for general practitioners, educational videos, a comprehensive website, an index card for intramural healthcare providers, and interactive educational sessions for healthcare providers. For each of these tools, a short design thinking process at microlevel was undertaken in the appointed supporting groups. Drawing upon the insights garnered from the extensive empathize and ideate phases mentioned above, brainstorming sessions in the relevant supporting groups defined the needs and desires for each tool and strengthened additional ideating. Iterative prototyping with refinement ensured that the tools aligned seamlessly with the objectives and requirements of the aimed rehabilitation pathway.

### Test

Testing of the rehabilitation pathway prototype is currently ongoing as part of a hybrid type 1 trial in a tertiary hospital setting to evaluate both real-world effectiveness and implementability (ClinicalTrials.gov NCT03427099). This is a nonrandomized controlled trial, where the prospective cohort of 36 patients receiving usual care (followed up during the initial empathize phase) serves as the control group. The intervention group includes patients undergoing single- or double level lumbar fusion surgery for degenerative conditions, and all healthcare providers involved in the management of these patients. The results of the testing phase, both the effectiveness and the implementability evaluation, will be presented in forthcoming research papers [60,61].

#### Effectiveness evaluation

Outcome indicators have been established according to the Leuven Clinical Pathway Compass, and in line with the quadruple aim to improve health outcomes, patient satisfaction, healthcare provider satisfaction and reducing costs (Table 3) [40,41]. Patients are followed up at baseline (start of the rehabilitation pathway), one day preoperatively and four days, six weeks, three months, six months, one year and two years postoperatively.

**Table 3:** Outcome indicators to evaluate the effectiveness of the rehabilitation pathway.


**Clinical indicators**	

Disability (primary outcome)	Oswestry Disability Index

Work resumption	Timing and percentage of work resumption

Back pain intensity	Numerical Pain Rating Scale

Leg pain intensity	Numerical Pain Rating Scale

Kinesiophobia	Tampa Scale for Kinesiophobia

Depression, anxiety, stress	Depression Anxiety and Stress Scale

Pain catastrophizing	Pain Catastrophizing Scale

Quality of life	EuroQoL-5D-3L

Sit-to-stand performance	Five times Sit-to-stand test (duration)

**Patient and team indicators**	

Part of the implementability evaluation	

**Financial indicators**	

Direct costs per patient	(1) Inpatient healthcare costs related to the hospitalization for lumbar fusion surgery; (2) Outpatient healthcare costs related to the lumbar fusion surgery; (3) Healthcare costs related to the rehabilitation

Indirect societal cost per patient	Sick leave

**Process indicators**	

Key rehabilitation interventions	Percentage based on nominator (number of patients receiving the rehabilitation intervention) and denominator (number of patients where the rehabilitation intervention is indicated)

Other process indicators	Part of the implementability evaluation


#### Implementability evaluation

Implementability is being evaluated iteratively in line with the Framework of Implementability of Healthcare Interventions [31]. The following constructs of implementability are being assessed: acceptability, fidelity, feasibility, and sustainability. Scalability will be estimated separately in the form of a within-trial cost-utility analysis. Additionally, implementation barriers/enablers were explored, and the reach of the implementation was captured. Data is provided by five sources: (i) healthcare provider and administrator surveys, (ii) patient surveys, (iii) file audit of patients’ records, (iv) independent fidelity checks, and (v) focus group of healthcare providers guided by the Theoretical Domains Framework. Patients are surveyed at baseline (start of the rehabilitation pathway), 6- and 12-months postoperative. File audits and surveys of healthcare providers and administrators are conducted at the start of the rehabilitation pathway, 6- and 12-months follow-up.

### The infinity component

During the testing phase, iterative feedback loops will inform refinements in the rehabilitation pathway and its implementation. Moreover, the testing phase allows us to learn more about the barriers and facilitators of implementing this rehabilitation pathway from the perspective of end-users and legislation, which will inform further refinements.

The steering committee and the supporting group prepared the implementation of the rehabilitation pathway, and continuously evaluate and improve this by additional loops of design thinking: learning about barriers and facilitators (empathize and define) ask for additional solutions (ideate, prototype and test).

## Results

The final prototype is named the ‘REACT rehabilitation pathway’ and is outlined in Table 4. A description following the Template for Intervention Description and Replication (TIDieR) [42] and a visual summary for patients are available in Appendix 1.

**Table 4:** Detailed description of the REACT rehabilitation pathway for patients undergoing lumbar fusion surgery.


**Pathway**	The REACT rehabilitation pathway begins with a preoperative intake, and continues up to one year postoperative. The case manager contacts the patient by telephone once the lumbar fusion surgery is scheduled to plan the preoperative intake consultations. Uniform communication of all healthcare providers involved is important.

***Pre**habilitation*	Patients receive a preoperative face-to-face intake of 30 minutes with a case manager (preferably with a background in Physical and Rehabilitation Medicine or musculoskeletal physiotherapy), who aims to serve as a contact and trust person during the REACT rehabilitation pathway. All patients receive the same educational leaflet and guidance to a website with educational videos. During the intake session, the content of the leaflet is discussed in a person-tailored manner. This focusses on education (including pain education), setting realistic goals and expectations, creating a therapeutic alliance, promoting a healthy lifestyle including smoking cessation, healthy diet, and psychosocial support. Any potential (biopsychosocial) barriers to the rehabilitation process are also discussed (including a psychosocial screening). Additionally, a 30-minute intake with specialized musculoskeletal physiotherapist, affiliated to the hospital is conducted (including education, teaching postoperative transfers, patient-specific ergonomic advice, encouragement of physical activity). Following these two preoperative intake consultations, the case manager and physiotherapist discuss any points of attention for the rehabilitation (other disciplines can be involved in the interdisciplinary discussion depending on the issues), and the physiotherapist contacts the treating (primary care) physiotherapist to explain the REACT rehabilitation pathway, and essential information for referral. Patients can choose their own physiotherapist. If the patient do not have a preferred physiotherapist, the case manager and specialized physiotherapist may suggest physiotherapists in their local area who are able to align with the philosophy of the REACT pathway. In case of specific issues noticed during the intake consultations, these are addressed, or the patient will be referred for preoperative therapy such as musculoskeletal physiotherapy, psychomotor therapy, psychological therapy or other guidance (e.g. smoking cessation).

***Peri**operative rehabilitation*	During hospitalization, early mobilization after lumbar fusion surgery is applied – getting the patient out of bed as soon as possible (no later than 24 hours postoperatively) and avoiding any unsubstantiated movement restrictions. This philosophy of focusing on early mobilization, positive empowerment, and being as liberal as possible regarding movement should be uniformly communicated by all involved healthcare providers and in the educational materials. Patients should receive daily physiotherapy perioperatively (including education, transfers, gait rehabilitation, doing stairs, advice for ADL and participation) that proceeds criteria based. Each patient is discussed interdisciplinary, and other healthcare providers (e.g. psychologist, occupational therapist) can be involved if indicated. Patients receive an X-ray before hospital discharge.

***Minimal restrictions***	Movements and activities with low to moderate axial loading are immediately allowed postoperatively (e.g. bending, rotating, typical household tasks, walking, cycling). Return to work is allowed as soon as feasible. High loading movements and activities (e.g. lifting heavy objects, impact sports) are restricted postoperatively during the first three months. Thereafter, no restrictions do apply. No postoperative bracing is advised.

***Post**operative rehabilitation*	Patients continue the rehabilitation with their treating physiotherapist (in primary care). It is advised to start within the first two weeks postoperatively. Physiotherapists will once again be contacted by the specialized physiotherapist (affiliated with the hospital) to provide additional patient-specific information that may have raised during perioperative rehabilitation and to ensure that the physiotherapy aligns with the REACT pathway. Physiotherapists are told that they can always contact the case manager or specialized physiotherapist in case of any questions or concerns that may arise during the initial and further stages of rehabilitation. Physiotherapy includes education, cardiovascular training, functional training of activities (including graded activity), optimization of participation, optimization of posture and movement control (with cognitive behavioral aspects, ergonomic advice, analyzing and treating maladaptive movement patterns, if indicated), and can be discontinued after reaching the goals set by the patient (e.g. specific household task, sport resumption). If indicated (e.g. specific psychosocial risk factors, complicated course), additional healthcare providers can be involved, or an interdisciplinary rehabilitation program affiliated to a hospital can be initiated.

***Case manager** follow-up*	Face-to-face follow-up consults with the case manager, lasting approximately 30 minutes, take place at four days postoperatively (or on the last day of a shorter hospitalization) and at six weeks, three months, six months, and one year postoperatively. During these consults, the case manager performs a person-centered history and physical examination, and apply a flexible clinical reasoning approach to evaluate all potential contributing factors to residual symptoms or activity limitations. Additionally, the case manager explores potentially interfering factors with rehabilitation across the biopsychosocial spectrum, and tries to validate potential concerns, provide reassurance regarding the expected course after lumbar fusion surgery and an understandable explanation of residual (or new) pain or symptoms. Medication will be also evaluated, and the rehabilitation plan will be optimized if necessary. Great emphasis should be placed on reassuring and empowering patients to progressively resume their daily activities, sports, and work. Any barriers that patients encounter in doing so should be mapped and addressed. Patients are actively stimulated and supported to resume work. Patients receive the contact information of the case manager (telephone and e-mail), and can contact the case manager if they have questions or concerns. An additional face-to-face consult with the case manager is possible in shared decision with the patient. If a complication or a structural cause of residual or new symptoms is suspected, the case manager will seek additional advice from the treating surgeon. At six weeks postoperatively, patients have a consultation with their treating surgeon. During this consultation, radiographs will be ordered only when clinically indicated. The case manager will oversee the rehabilitation pathway and initiate interdisciplinary discussions when needed.


## Discussion

This paper describes the development of a user-centred and evidence-based rehabilitation pathway for lumbar fusion surgery by the adoption of a design thinking framework.

This rehabilitation pathway aims to provide guidance on the optimal rehabilitation for patients undergoing lumbar fusion surgery. Although the importance of rehabilitation in improving clinical outcomes after lumbar fusion surgery has been demonstrated in prior research over the last two decades, this evidence was not effectively translated into daily practice (‘translational gap’), and uncertainties concerning the optimal timing and content persisted (‘knowledge gap’) [7,10,11,12,13]. The use of design thinking facilitated the efforts to address the knowledge gaps and enhance the ability to bridge the translational gap.

### Clinical innovation

This rehabilitation pathway intends to serve as a unifying guideline for patients undergoing lumbar fusion surgery, across disciplines, across care settings, and across the entire time-continuum of care (i.e., pre-, peri- and postoperatively).

The introduction of prehabilitation, which is also gaining interest in the context of other surgical procedures, sets the stage for a proactive approach that empowers patients early on as active participants in their own care [43]. In this proactive approach, a case manager plays a key role as a central point of contact and a person of trust. Prehabilitation can give patients a head start for a better postoperative recovery after lumbar fusion surgery by focusing on education, intake of physiotherapy and interventions to address potential risk factors. In doing so, this pathway expands on the existing evidence base, where risk factors for suboptimal postoperative recovery have been identified, but therapeutic implications were limited [44,45,46,47,48,49,50]. By emphasizing early postoperative mobilization, consistent with the Enhanced Recovery After Surgery recommendation [51], the pathway encourages patients to start moving as soon as possible after surgery. In the postoperative phase, the rehabilitation pathway promotes a rapid return to functional activities by allowing low to moderate axial loading immediately after surgery, by not prescribing postoperative braces, and by scheduling radiographs only when clinically indicated. This contrasts with current practices that often involve strict activity restrictions [12], postoperative bracing [13], and routine radiographs [52] unsupported by robust evidence.

Importantly, this rehabilitation pathway offers a standardized, yet flexible pathway, that may be tailored to individual patient needs. While rehabilitation, by its very nature, is a highly patient-centred strategy, the possibility to tailor rehabilitation was often absent in previously developed rehabilitation interventions [7].

### Methodological innovation

Empathy for end-users forms the centrepiece of design thinking [21]. As researchers, the problems we try to solve are rarely our own, which can possibly lead to a mismatch between the research focus and the actual needs of end-users. Despite increasing encouragement to involve patients as partners in recent years, this remains the exception in clinical pain research [53].

The “empathize” phase revealed crucial insights that shaped the development of the rehabilitation pathway. For instance, the problem statement was redefined when it became evident that the rehabilitation process should encompass the entire patient journey and not only the postoperative phase. Additionally, the rehabilitation pathway needed to be standardized across disciplines, involving the primary care setting as well. The empathetic approach revealed that some healthcare providers felt undervalued in interdisciplinary meetings. To mitigate potential authority bias, we also included monodisciplinary iterations during prototyping, aiming to ensure equal value for all perspectives.

Incorporating robust research methodologies, such as a systematic review with meta-analysis and a modified Delphi study, into the design thinking framework, aimed to strengthen the evidence-based foundation of the resulting rehabilitation pathway.

Although it might seem intuitive to utilize current evidence when designing new interventions, this is often not the case [54]. Worryingly, previous research has revealed that less than half of researchers acknowledge being aware of relevant reviews regarding existing evidence while designing their clinical trials [55], and that even in highly cited medical journals, researchers seldom indicated the incorporation of recent systematic reviews into their trial design [54]. Neglecting established evidence may result in unfounded research and wasted efforts, further contributing to the issue of global research waste. In an era of increasing investment in biomedical research, which reached $240 billion USD in 2010, the need to reduce (avoidable) research waste is imperative. Research waste is reported to emerge when future users’ needs were ignored (lack of empathy), and what we already know or have studied was overlooked (lack of evidence review) [54].

Design thinking is a replicable method that could be useful in different contexts. Our paper’s strength lies in the transparent and rigorous reporting of our design thinking process. This can guide researchers in the adoption of design thinking for development of rehabilitation pathways for other types of surgery or health conditions. The World Health Organization (WHO) has recognized the increasing global need for rehabilitation, and prioritized it through the ‘Rehabilitation 2030: a call to action’ initiative, which aims to promote well-being and healthy lives [56]. With the potential to benefit a substantial portion of individuals living with disability, addressing this rehabilitation need is critical [56]. Researchers are urged to fill the knowledge gaps in rehabilitation [56,57]. Developing real-world rehabilitation interventions has methodological challenges due to a dynamic and variable environment, unlike the constant enclosed environment of, for example, a petri dish for developing new antibiotics [51,52,53]. Design thinking, with its user-centred and flexible approach, has the potential to adapt to the ever-changing healthcare landscape [21,58].

Future directions include testing the rehabilitation pathway in a hybrid type 1 trial [59]. Such a hybrid design will evaluate the effectiveness and the implementability of the rehabilitation pathway.

A cost-utility analysis should be an essential part of the effectiveness evaluation. During the ideate phase of our pathway, no financial constraints were considered, which allowed for creative and out-of-the-box ideas. During the prototype phase, some financial barriers were considered to improve the potential for implementation.

To improve the implementation potential, a final recommendation is to involve health insurers in future refinements. The infinity aspect, or ‘design-post-design’, of design thinking makes it possible to do so.

## Conclusion

Design thinking, exemplified by the infinity loop, guided the development of an integrated rehabilitation pathway. Prioritizing an in-depth understanding of end-user needs enhanced both the relevance and feasibility of the resulting pathway in a real-world context. Additionally, the integration of rigorous research methods strengthened the evidence base of the final pathway. The transparent application of design thinking in this study may inform the development of future rehabilitation pathways addressing other healthcare conditions.

## Additional File

The additional file for this article can be found as follows:

10.5334/ijic.7765.s1 Appendix 1. Description of the REACT rehabilitation pathway (TIDieR) and visual patient summary.
