# Supplementary material for: The Infinity Loop of Healthcare Innovation: Development of an Integrated Rehabilitation Pathway for Lumbar Fusion Surgery Through Design Thinking
Source: Int J Integr Care. 2025 May 12;25(2):11. doi: 10.5334/ijic.7765 (PMC12082458; doi:10.5334/ijic.7765)
Supplement: Appendix 1. — Description of the REACT rehabilitation pathway (TIDieR) and visual patient summary. [file ijic-25-2-7765-s1.pdf]

Appendix 1

TIDieR item	Contents
**Brief name**	**Case manager guidance**
**Why (rationale)**	A case manager will contribute to uniform and tailored information to the patient, to a lower barrier for patients to contact a healthcare provider with questions or problems, and to better interdisciplinary and transmural collaboration.
**What**	A case manager will be appointed for the pre-, peri- and postoperative rehabilitation process, and will be the contact person for patients with questions or problems and can involve other healthcare providers if necessary
**Who (profession)**	This contact person has the right competence profile with sufficient biomedical and psychosocial knowledge. E.g., a physiotherapist, a specialist in physical medicine and rehabilitation
**How (modes of delivery)**	Face-to-face, by telephone or by email. All contact moments are individual.
**Where (infrastructure)**	All the face-to-face contact moments are conducted at the hospital.
**When, how much (number of sessions, duration, intensity or dose)**	A preoperative intake of 30 minutes, postoperative consultations of 30 minutes at four days, six weeks, three months, six months and one year postoperatively.
**Tailoring (personalization)**	Additional contact moments (face-to-face, by telephone or by email) are possible based on the needs of the patients

TIDieR item	Contents
**Brief name**	**Prehabilitation**
**Why (rationale)**	Starting the rehabilitation process before the surgery is an opportunity to take a head start in the rehabilitation, to set realistic expectations, to create a therapeutic alliance, and to tackle potential risk factors which may have an adverse effect on postoperative recovery and outcomes.
**What**	Educational materials:<br>○ patient leaflet of 30 pages outlining education regarding the following topics: rationale and process of designing the rehabilitation pathway, indication and procedure of lumbar fusion surgery, importance of rehabilitation, content of their personalized rehabilitation pathway, optimal preparation for their surgery, day of surgery, what to expect immediately postoperative, progressive build-up of activities after their hospitalization, follow-up after hospitalization, return to work, and contact information.<br>○ Educational website (https://www.uzleuven.be/nl/wervelkolomaandoeningen/lumbale-fusie-op-1-2-niveaus and https://www.uzleuven.be/nl/react)<br>○ Twelve short educational videos (available via the website) where different healthcare providers involved in the rehabilitation pathway answer highly relevant and frequently asked questions regarding the rehabilitation<br>Procedures:<br>○ Preoperative physiotherapeutic intake including education, teaching postoperative transfers, patient-specific ergonomic advice, encouragement of physical activity.<br>○ Preoperative intake with case manager focused on education, setting realistic goals and expectations, creating a therapeutic alliance, promoting a healthy lifestyle including smoking cessation, healthy diet, and healthy mindset.<br>○ Preoperative intra- (between physiotherapists) and interdisciplinary discussion
**Who (profession)**	Physiotherapist experienced in spinal rehabilitation, Case manager with sufficient biopsychosocial knowledge.
**How (modes of delivery)**	Face-to-face, individual<br>Educational materials on paper (leaflet) and online
**Where (infrastructure)**	Face-to-face intake session is provided at the hospital.
**When, how much (number of sessions, duration, intensity or dose)**	One preoperative intake of 60 minutes (30 minutes case manager, and 30 minutes physiotherapist).
**Tailoring (personalization)**	Additional physiotherapeutic (in primary care or ambulant in the hospital) and/or other treatments (e.g., psychological support, support with smoking cessation) are possible based on the individual needs of the patients and the assessment during the interdisciplinary discussion

TIDieR item	Contents
**Brief name**	**Early postoperative rehabilitation during hospitalization**
**Why (rationale)**	Starting postoperative mobilization, education and functional training as soon as possible following surgery will accelerate the recovery
**What**	To facilitate early mobilization, lumbar fusion surgery is planned in the beginning of the week if no weekend physiotherapy is available in the hospital. In our case, weekend therapy is available.<br>Early mobilization (as soon as possible) following surgery, and daily physiotherapy, including education, functional movements (standing up, transfers, gait rehabilitation, climbing stairs), patient-specific ergonomic advice, encouragement of physical activity.<br>A formal interdisciplinary discussion to assess the postoperative needs.<br>Discharge criteria are controllable pain, clean wound, basic ADL, stairs (if necessary, in home situation)
**Who (profession)**	Physiotherapists and nurses experienced in spinal rehabilitation.<br>Education on early mobilization is supported by all involved care givers
**How (modes of delivery)**	Face-to-face, individual
**Where (infrastructure)**	During hospitalization
**When, how much (number of sessions, duration, intensity or dose)**	Each day of hospitalization at least one session of physiotherapy
**Tailoring (personalization)**	Additional healthcare providers can be involved on indication (e.g., psychomotor therapist, psychologist, ergotherapist)<br>Interdisciplinary discussion between physiotherapist, surgeon, nurse, ergotherapist and specialist in physical medicine and rehabilitation

TIDieR item	Contents
**Brief name**	**Continuation of postoperative rehabilitation after hospitalization**
**Why (rationale)**	Continuation of postoperative rehabilitation after hospitalization with their physiotherapist in primary care will optimize their recovery
**What**	Physiotherapy including education, cardiovascular training, functional training of activities, optimization of participation, optimization of posture and movement control (with or without cognitive behavioral aspects, ergonomic advice, analyzing and treating maladaptive movement patterns).<br>Follow-up by their general practitioner at 2 weeks postoperative, and at least one follow-up by their surgeon around 6 weeks postoperative. Follow-up by their case manager up to one year postoperatively.<br>No standard X-Rays are planned. No postoperative bracing is advised. No restrictions apply towards low to moderate axial loading. High loading (heavy lifting, certain contact/impact sports) is allowed from 3 months onwards.
**Who (profession)**	Skilled physiotherapist in primary care setting.<br>General practitioner, treating surgeon and appointed case manager.
**How (modes of delivery)**	Face-to-face, individual . Additional individual contact with case manager via telephone or email is possible (or face-to-face if needed based on the assessment of the case manager).
**Where (infrastructure)**	In primary care setting.<br>Follow-up appointments with the surgeon and the case manager are at the hospital.
**When, how much (number of sessions, duration, intensity or dose)**	Number and frequency of sessions with the physiotherapist are determined based on the need (up to 60 sessions possible), one session has a duration between 30 and 45 minutes. Follow-up are scheduled at 2 weeks (general practitioner), around 6 weeks (surgeon together with case manager), 3 and 6 months (case manager) and 1 year postoperatively (surgeon together with case manager).
**Tailoring (personalization)**	On indication, additional healthcare providers can be involved (e.g., psychologist) or an interdisciplinary rehabilitation program affiliated to a hospital can be started.
